# Intraprocedural and Delayed Plaque Protrusion in Carotid Artery Stenting Using a Dual-Layer Metallic Stent

**DOI:** 10.1016/j.jscai.2024.102285

**Published:** 2024-10-29

**Authors:** Kaoru Myouchin, Katsutoshi Takayama, Takeshi Wada, Yuto Chanoki, Hayato Kishida, Takahiro Masutani, Yumi Ko, Yoshitomo Uchiyama, Ichiro Nakagawa, Toshihiro Tanaka

**Affiliations:** a Department of Interventional Neuroradiology/Radiology, Kouseikai Takai Hospital, Tenri, Japan; b Department of Diagnostic and Interventional Radiology, Nara Medical University, Kashihara, Japan; c Department of Neurosurgery, Ishinkai Yao General Hospital, Yao, Japan; d Department of Neurosurgery, Nara Medical University, Kashihara, Japan

**Keywords:** carotid artery stenting, delayed plaque protrusion, dual-layer metallic stent, plaque protrusion

## Abstract

**Background:**

Intravascular ultrasound–determined plaque protrusion (PP) during carotid artery stenting (CAS) using conventional stents is reported in 7.6% to 12% of cases and is associated with periprocedural cerebral embolism. The Casper/Roadsaver stent (CRS) is a dual-layer micromesh stent designed to reduce the risk of PP, with a mesh cell diameter 4-fold smaller size than that of conventional stents. This study investigated the incidence of PP with CRS CAS.

**Methods:**

We prospectively analyzed 89 consecutive arteriosclerotic carotid artery stenoses in 82 patients (64 men; mean age, 76.8 years; 43 symptomatic) who underwent CAS with CRS under intravascular ultrasound. The main end points were the technical success rate, incidences of intraprocedural PP and at 1 week after CAS (delayed PP), incidence of new ipsilateral diffusion-weighted imaging lesion within 48 hours post CAS, and major adverse events (myocardial infarction, stroke, death) within 30 days. Secondary end points were the rate of in-stent restenosis and ipsilateral stroke at 30 days and 12 months.

**Results:**

The technical success rate was 100%. Intraprocedural PP occurred in 2 patients (2.2%). Delayed PP occurred in 3 additional patients (3.4%). Diffusion-weighted imaging positivity was 24.7%. Major adverse events (minor stroke) occurred in 1 patient (1.1%). In-stent restenosis occurred in 5 patients (6.0%) by 12 months. No ipsilateral stroke occurred during the follow-up.

**Conclusions:**

The incidence of intraprocedural PP with CRS CAS was 2.2%, indicating a significant reduction compared to conventional stents. However, at 7 days new PP had occurred in 3.4% of patients, indicating that patients with CRS should be followed up for delayed PP.

## Introduction

Risk factors associated with periprocedural ischemic complications during carotid artery stenting (CAS) include lack of a protection device, poor operator skills, patient age, plaque characteristics, stent design, and plaque protrusion (PP) during CAS.^1^, ^2^, ^3^, ^4^, ^5^, ^6^ Single-layer closed-cell design may somewhat reduce PP in relation to an open-cell design,^7^ but the incidence of intravascular ultrasound (IVUS)-detected PP during CAS using conventional single-layer stents may still be as high as 12%.^8^ The dual-layer micromesh Casper/Roadsaver stent (CRS) (Terumo Clinical Supply Co) has a much smaller free cell diameter (375-500 μm) than single-layer closed- and open-cell stents (1000-1900 μm), and is already in clinical use in Europe and Japan, with recent studies finding that it reduces the incidence of periprocedural ischemic complications compared with conventional stents.^9^, ^10^, ^11^, ^12^, ^13^ The aim of this study was to examine the incidence of intraprocedural PP and as well as PP at 7 days (delayed PP) in CAS using CRS.

## Methods

This study prospectively analyzed 82 consecutive patients (mean age, 76.8 years; range, 54-94 years; 64 men) with atherosclerotic carotid stenosis (89 stenoses) who underwent CAS under IVUS at our hospital or an affiliated institution between October 2020 and September 2023.

The study was approved by the research ethics committee of each participating hospital. Written informed consent was obtained from all patients prior to enrollment in this study.

### IVUS

A Volcano Visions PV 0.014P IVUS catheter (Volcano Corp.) was used. Combined analysis using gray-scale and ChromaFlo IVUS was used to detect the plaque lumen and stent/lumen border.^14^

### Magnetic resonance plaque imaging

Magnetic resonance (MR) imaging for plaque characterization was performed using an NT Intera 1.5-T POWER scanner or ACHIEVA 3.0-T Quasar scanner (Philips Medical Systems), MAGNETOM Prisma 3.0-T Power scanner (Siemens), or OPTIMA 450w 1.5-T Power scanner (GE Medical Systems). A 2-dimensional T1-weighted fast spin-echo sequence was employed using a black-blood, double inversion recovery preparation pulse, and a fat-saturation pulse. Signal intensities of the carotid plaque were evaluated at the level of most severe stenosis. Soft/unstable plaque was defined as having an average relative overall signal intensity >1.25 compared to that in the sternocleidomastoid muscle.^15^

### Diffusion-weighted imaging

Diffusion-weighted imaging (DWI) was performed using MR scanners with field strengths of 1.5 or 3.0 T operating. The imaging protocol involved DWI sequences with the following characteristics (*b* = 0, 1000 s/mm^2^; section thickness, 5.0 mm; gap, 1 mm). Apparent diffusion coefficient maps were obtained in all cases. The protocol specified that MR scans were to take place within 72 hours before CAS and within 48 hours after CAS. Two board-certified neuroradiologists (K.T. and T.W.) independently analyzed all scans in a blinded manner.

### Ultrasound

Cervical color and power Doppler US were performed at 1, 3, and 7 days after CAS, and then at 1, 3, 6, and 12 months. The examination used a color duplex ultrasound system (model Aplio i700; Canon Medical Systems Corp.) with a probe frequency of 7.5 MHz.

### Definition of PP

Intraprocedural PP was defined as the presence of any material other than that suggestive of thrombus inside the inner mesh luminal border on IVUS immediately after postdilatation.

Delayed PP was defined as in-stent plaque material other than that suggestive of thrombus, as evaluated on the cervical US within 1 week after CAS.

### Definition of in-stent restenosis

In-stent restenosis (ISR) was defined as stenosis reducing the vessel diameter by ≥50% or peak systolic velocity ≥170 c m/s^16^ by the cervical US.

### CAS procedure

All patients started dual-antiplatelet therapy (aspirin 100 mg/d and clopidogrel 75 mg/d) at least 1 week before CAS. Patients were continued on dual-antiplatelet therapy for at least 6 months.

Unfractionated heparin was administered during the procedure to maintain an activated clotting time >250 seconds. All CAS procedures were performed under local anesthesia using dual embolic protection (Mo.Ma Ultra; Medtronic or OPTIMO; Tokai Medical Products, and Filterwire EZ; Boston Scientific Corp.) unless contraindicated by intolerance to ischemia or anatomical infeasibility; in such cases, the Filterwire EZ alone was employed. Access was transfemoral, transradial, or transbrachial as decided by the operator in consideration of the anatomic conditions of the patient.

Protected CAS was performed after predilatation with a 3- or 4-mm balloon (predilatation was not performed in cases with ≤60% stenosis) and deployment of a CRS followed by conservative postdilatation using an angioplasty balloon sized ∼80% of the normal lumen diameter just distal to the stenosis, as determined by IVUS.

### Outcomes measures and definitions

The main end points of this study were the technical success rate, incidences of intraprocedural PP and delayed PP, rate of ipsilateral ischemic lesions on DWI within 48 hours after CAS, and rate of major adverse events (myocardial infarction, stroke, or death) within 30 days. Technical success was defined as a minimum stent lumen diameter ≥3 mm on IVUS after postdilatation or residual stenosis <30% of vessel diameter on digital subtraction angiography (DSA). Stroke was defined as ischemic neurological deficit persisting >24 hours. Stroke severity was classified using the National Institutes of Health Stroke Scale (NIHSS) and the modified Rankin Scale (mRS) at 30 days post CAS. Major stroke was defined as an NIHSS score >5 or mRS score >2, whereas minor stroke was classified as an NIHSS score ≤4 and mRS score ≤2.

Secondary end points were the rate of ISR and ipsilateral stroke at 30 days to 12 months.

## Results

A total of 82 patients were enrolled in this study, with 43 patients (48.3%) identified as symptomatic. Symptoms in these 43 patients comprised transient ischemic attack in 7 patients (16.0%) and minor stroke in 36 patients (84.0%) of the symptomatic cohort. Fifty-three stenoses (59.6% including 32 [73%] in symptomatic patients) showed unstable plaque characteristics on MRI. The mean degree of stenosis according to the North American Symptomatic Carotid Endarterectomy Trial method was 81% (range, 50%-99%). Baseline characteristics of the study patients are summarized in Table 1. A transfemoral approach was applied for 82 stenoses (92.0%), transradial approach for 6 (7.0%), and transbrachial approach for 1 (1.0%). Protection comprised distal filter alone for 8 stenoses (9.0%), and filter and balloon-guided catheter for 81 (91.0%). For these 81 cases, the balloon-guided catheter was an OPTIMO for 45 stenoses whereas the Mo.Ma Ultra was used for 36 cases of stenoses.

**Table 1:** Demographics and baseline characteristics of patients included in the prospective analysis.

Characteristic	N = 82
Age, y	76.8 ± 8.1
Male sex	64 (78.0%)
Hypertension	71 (86.6%)
Hyperlipidemia	60 (73.2%)
Diabetes mellitus	32 (39.0%)
Cigarette smoking (current)	17 (20.7%)
Previous myocardial infarction	29 (35.4%)
Stroke/transient ischemic attack within 6 months	43 (48.3%)
Femoral access	82 (92.1%)
Radial access	6 (6.7%)
Brachial access	1 (1.1%)
Target vessel	
Stenosis, %	81.0 ± 13.8
Right internal carotid artery	45 (50.6%)
Left internal carotid artery	41 (46.1%)
Right common carotid artery	2 (2.2%)
Left common carotid artery	1 (1.1%)
Lesion length, mm	21.4 ± 16.3
Protection used	
Distal filter protection alone	8 (9.0%)
Proximal balloon protection and distal filter protection	81 (91.0%)
Predilatation	84 (94.4%)
Postdilatation	89 (100%)
Postdilatation diameter, mm	4.08 ± 0.58

Values are mean ± SD or n (%).

**Figure 1:**
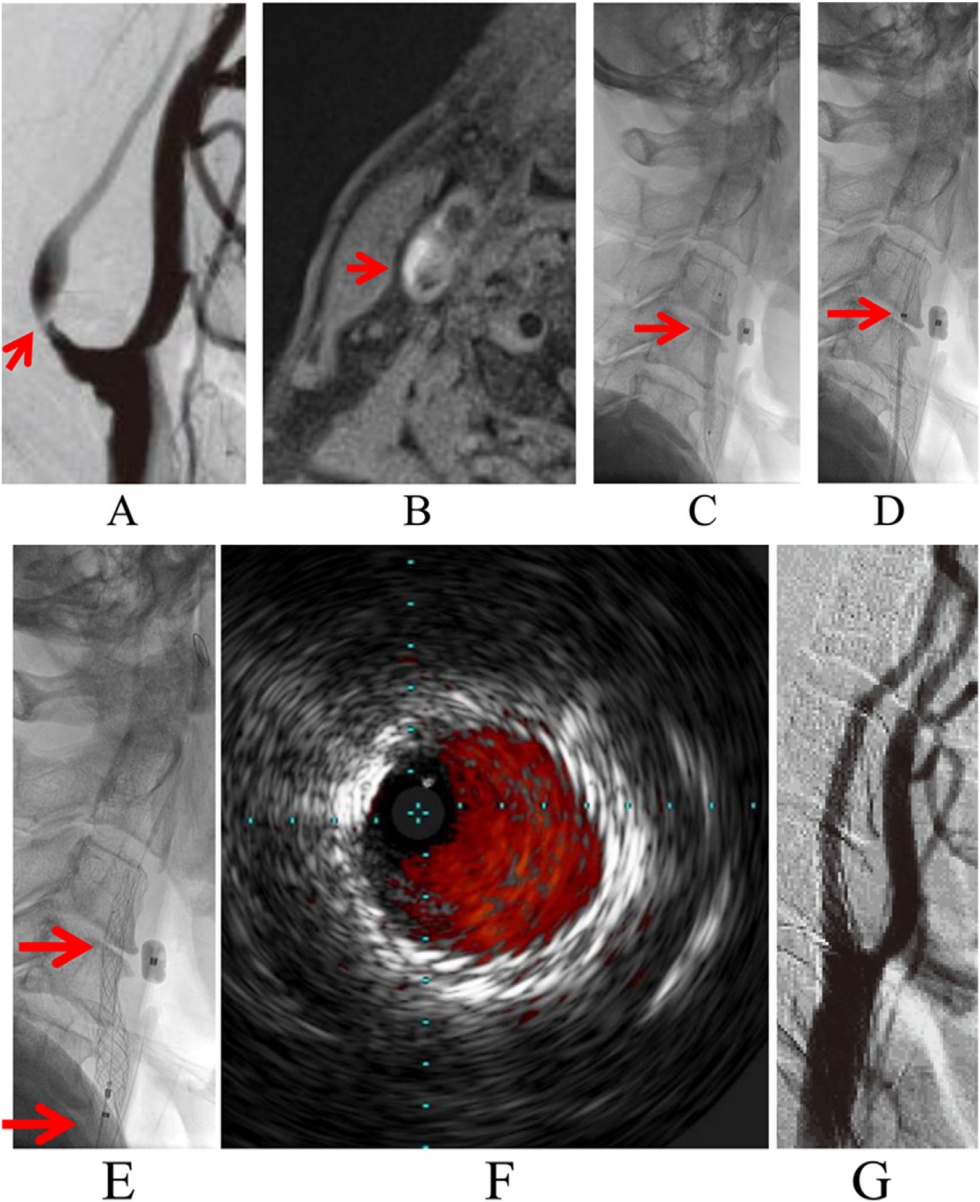
**A case of intraprocedural plaque protrusion.** (**A**) Angiogram of the right common carotid artery (lateral view). Severe stenosis is evident at the origin of the right internal carotid artery (arrow). (**B**) Preoperative MRI of the plaque with a 3-T scanner and 2-dimensional T1-weighted black-blood method. A high-intensity signal is evident at the origin of the right internal carotid artery (arrow), suggesting the presence of unstable plaque. (**C**) Live image of the right common carotid artery (lateral view). Postdilatation was performed with a balloon catheter (4 mm × 40 mm) (arrow) after Casper/Roadsaver stent (CRS) placement under proximal protection provided by the Mo.Ma Ultra and filter protection by the Filterwire EZ. (**D**) Live image of the right common carotid artery (lateral view). A second stent (CRS 8 mm × 20 mm) is guided inside the first stent because intravascular ultrasound after postdilatation shows apparent plaque protrusion (see Central Illustration panel A). (**E**) Live image of the right common carotid artery (lateral view). The second stent (CRS 8 mm × 20 mm) is deployed inside the first stent. (**F**) Intravascular ultrasound at the minimum lumen diameter of the lesion site after the second stent placement confirms the absence of plaque protrusion. (**G**) Right common carotid angiogram (lateral view). The final angiogram shows good dilation at the stenotic site with no obvious plaque protrusion.

Procedure characteristics are summarized in Table 1. The technical success rate was 100% (Table 2). Intraprocedural PP occurred in 2 patients (2.2%) (Figure 1 and Central Illustration).^17^ In one of those 2 cases, the patient showed asymptomatic PP immediately after postdilatation and was treated with the stent-in-stent technique using another CRS in the same session, as the degree of PP was deemed substantial with a high risk of ischemic complications. The other case showed protrusion of less than one-fourth of the stent circumference and less than 1 mm into the lumen. This protrusion was only visible on IVUS, and not on angiography. After 10 minutes, PP showed no change on IVUS, and the decision was made to perform a follow-up. The patient remained asymptomatic and DWI the next day showed no new lesions. Follow-up US was performed at 1, 3, and 7 days after CAS consistently. There was no evidence of maintained PP and no ischemic cerebral symptoms occurred.

**Table 2:** Summary of primary and secondary end points.

End point	N = 89
Technical success^a^	89/89 (100%)
Intraprocedural plaque protrusion	2/89 (2.2%)
Delayed plaque protrusion	3/89 (3.4%)
Ipsilateral ischemic lesions on DWI rate	22/89 (24.7%)
Major adverse events within 30 d	1/89^e^ (1.1%)
In-stent restenosis^c^	5/84 (6.0%)
Target lesion revascularization	4/84^b^ (4.8%)
Ipsilateral stroke after 30 d^d^	0/82 (0%)

DWI, diffusion-weighted imaging.

a A minimum stent lumen diameter ≥3 mm on intravascular ultrasound after postdilatation or residual stenosis <30% of vessel diameter on digital subtraction angiography.

b One patient refused additional treatment.

c Detected by follow-up cervical ultrasound at a mean of 15 months (range, 1-38 months).

d Mean of 18 months (range, 1-38 months).

e One minor stroke.

**Central Illustration:**
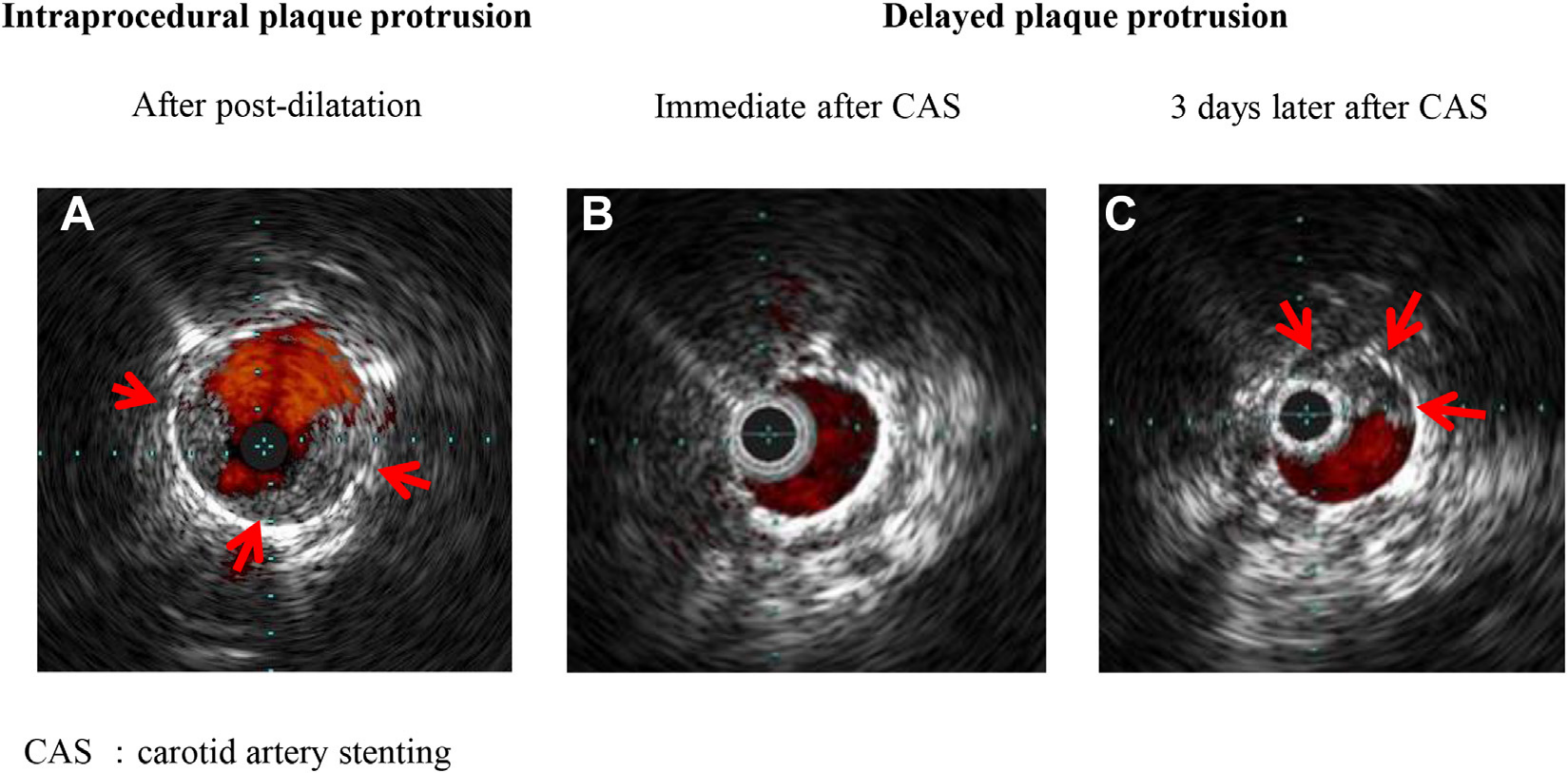
**Intraprocedural plaque protrusion and delayed plaque protrusion in carotid artery stenting (CAS).** (**A**) Intraprocedural plaque protrusion immediately after postdilatation; intravascular ultrasound shows plaque protrusion (arrows). (**B**) Delayed plaque protrusion immediately after postdilatation; intravascular ultrasound shows no evidence of plaque protrusion. (**C**) Delayed plaque protrusion 3 days after CAS; intravascular ultrasound shows plaque protrusion (arrows).

**Figure 2:**
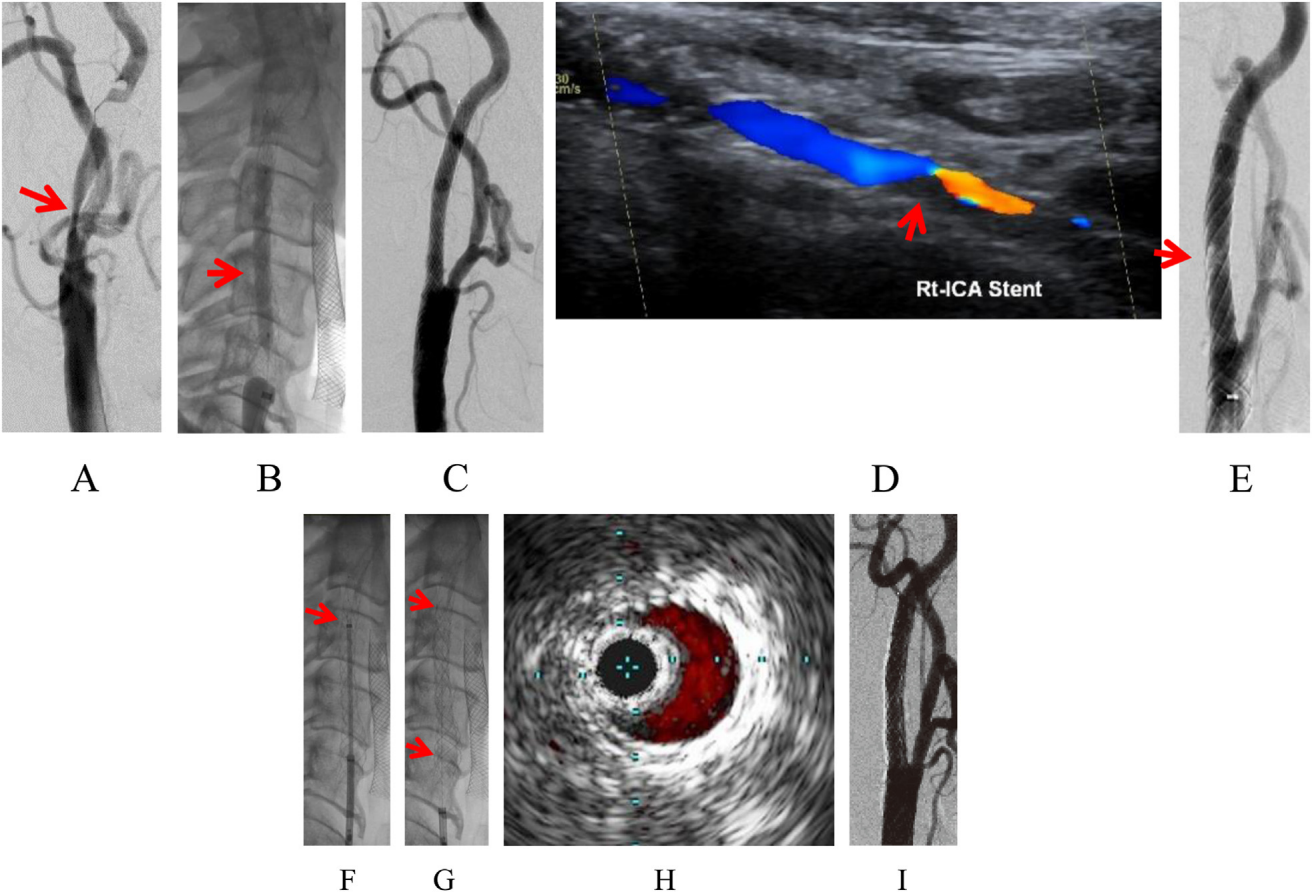
**A case of delayed plaque protrusion.** (**A**) Angiogram of the right common carotid artery (frontal view). Severe stenosis is seen at the origin of the right internal carotid artery (arrow). (**B**) Live image of the right common carotid artery (lateral view). Carotid artery stenting (CAS) using the Casper/Roadsaver stent (CRS) (8 mm × 30 mm) with postdilatation performed using a balloon catheter (4 mm × 40 mm) with OPTIMO and Filterwire EZ to treat internal carotid artery stenosis with unstable plaque. (**C**) Angiogram of the right common carotid (lateral view). The final angiogram shows good dilatation at the stenotic site, with no obvious plaque protrusion. Intravascular ultrasound at the minimum lumen diameter of the lesion site after stent placement does not show any plaque protrusion. (**D**) Ultrasound 3 days after CAS shows plaque protrusion (arrow). (**E**) Angiogram of the right common carotid artery 3 days after CAS (lateral view). Plaque protrusion is evident (arrow). (**F**) Live image of the right common carotid artery (lateral view). A second stent (CRS 6 mm × 30 mm) is guided inside the first stent because intravascular ultrasound at the minimum lumen diameter of the lesion site 3 days after CAS shows plaque protrusion. (**G**) Live image of the right common carotid artery (lateral view). The second stent (CRS 6 mm × 30 mm) is positioned in a stent-in-stent manner (arrows). (**H**) Intravascular ultrasound at the minimum lumen diameter of the lesion site after the second stent placement confirms the absence of plaque protrusion. (**I**) Angiogram of the right common carotid (lateral view). The final angiogram shows good dilatation at the stenotic site, with no obvious plaque protrusion.

Delayed PP was observed in 3 additional patients (3.4%) (Table 2), occurring 1 day after CAS in 1 patient and 3 days after CAS in 2 patients (Figure 2 and Central Illustration). All 3 patients with delayed PP had an unstable plaque on baseline MRI.

Two of the 3 patients with delayed PP were treated with the stent-in-stent technique, using a Carotid Wallstent (Boston Scientific) 2 days after CAS in one patient and using a CRS 3 days later after CAS in the other. The remaining patient received the best medical treatment with close monitoring by US, showing the disappearance of PP by 4 days after CAS. The rate of new ipsilateral ischemic lesions on DWI was 24.7%, with symptoms in 1 case. Major adverse events occurred in 1 patient (1.1%; minor stroke) (Table 2).

Follow-up cervical US (mean, 15.0 months; range, 1-38 months) was obtained in 77 patients with 84 stenoses (94%). By 12 months, ISR had occurred in 5 patients (6.0%). All 5 cases of ISR showed high-grade (≥80%) restenosis (99% at 3 months in 4 cases, 80% at 6 months in 1 case). However, ISR was asymptomatic in all 5 patients. Target lesion revascularization (TLR) was performed in 4 patients (4.8%) with additional stent placement in 3 cases and balloon angioplasty using a plain (ie, non–drug-eluting) balloon in 1. One patient declined additional treatment. TLR was performed without any complication.

Twelve-month clinical data were available for all study patients. No ipsilateral stroke occurred between 30- days and 12 months (mean follow-up of 18 months; 1-38 months) in any of the 82 patients (Table 2).

## Discussion

In a study of 328 patients undergoing CAS, Kotsugi et al^4^ documented a 7.6% PP rate with IVUS and 2.6% with angiography during CAS, identifying a strong association between intraprocedural PP and periprocedural cerebral infarction. The same study also identified unstable plaque and the use of an open-cell stent as risk factors for intraprocedural PP.^4^

Our data using a CRS showed a 2.2% incidence of IVUS-determined intraprocedural PP. This rate is substantially lower than that previously reported for the conventional (single-layer) carotid stent (7.6%-12%).^4^^,^^8^

In the present study, intraprocedural PP was observed in 2 cases. One of those 2 cases was evaluated by IVUS alone. Because the volume of PP seemed large enough to warrant concern for IVUS, we placed an additional stent to avoid ischemic complications without performing angiography. PP can be detected more frequently by IVUS than by DSA,^4^^,^^18^ consistent with the high-resolution 3-dimensional insight into the lumen provided by IVUS compared to the 2D presentation with DSA.

Yamada et al^19^ used optical coherence tomography (OCT) to investigate 46 consecutive patients who underwent CAS for carotid artery stenosis. That imaging modality offers even greater resolution than IVUS. A comparison between CRS and conventional stents found that the incidence of PP was significantly lower with CRS (44%) than with conventional stents (88%, *P* = .022). These results, using different imaging techniques, appear to be consistent in showing an over 2-fold reduction in PP incidence with CRS compared to conventional (single-layer) carotid stents. The 2 key findings are the incidence of “any” PP (and its evaluation on a per-patient or per-slice/per-segment basis), and the extent of PP (in terms of circumferential area and “depth,” which may confer different risks of cerebral embolism).^20^ In a recent nonrandomized study, CRS was associated with a lower incidence of embolism and lower filter load than conventional carotid stents.^21^ In a randomized controlled study by Karpenko et al,^22^ the CGuard MicroNet-covered stent (CGS) (InspireMD) significantly reduced periprocedural and abolished postprocedural DWI cerebral embolism in relation to a conventional carotid stent. DWI, used in our and other studies, provides the net effect of CAS-related embolic load to the brain that incorporates the fundamental stent type role among other procedural factors. In our study, the rate of new ipsilateral ischemic lesions on DWI was 24.7%, lower than that reported in a previous study using conventional stents.^7^^,^^23^ A systematic review and meta-analysis^24^ demonstrated a correlation between silent DWI lesions and the risk of clinically manifest stroke in carotid revascularization procedures. Given these findings, the CRS might be recommended over conventional (single-layer) stents.

The CRS seems to have disadvantages in terms of higher rates of restenosis and TLR compared with conventional stents. A systematic review and meta-analysis by Mazurek et al^25^ found that CRS showed a higher restenosis rate (7.16%) than conventional stents (3.97%). Although ISR and delayed PP seem to represent distinct phenomena, ISR may be associated with delayed PP. ISR in first-generation carotid stents is generally believed to be associated with an exaggerated healing response in the form of intimal hyperplasia formation, but 1 case report showed that restenosis may be underlined by atherosclerotic plaque progression via single-layer stent struts.^26^ One reason for this may be that single-layer stents may be associated with delayed PP, and our results showed a higher rate for delayed PP than for intraprocedural PP. In fact, the ISR rate in our study was 6.0%. Three of the 4 patients with delayed PP were treated with additional interventions. If those cases had not undergone additional interventions, ISR may have developed. Overall, the CRS ISR rate in our study is consistent with previous work.^25^ Matsumoto et al^27^ provided mechanistic insights into CRS ISR in 30 consecutive patients. They have indicated that dissociation between the 2 CRS metallic layers, observed on IVUS in 12 out of 30 patients (40%) may enhance CRS ISR. Our study also yielded findings consistent with those of Matsumoto et al^27^ in 2 (50%) of the 4 patients with ISR. These data may be indicative of a higher ISR rate with CRS than with conventional stents.

Mazurek et al^28^ conducted a meta-analysis of CEA outcomes for CAS with second-generation stents, revealing that CRS reduced 30-day and 12-month ipsilateral stroke rates, but increased the restenosis rate. On the other hand, CGS reduced both stroke and restenosis rates. CGS may thus be a clinically more favorable stent than CRS.

Delayed PP seemed related to in-stent thrombosis and ischemic stroke within 30 days. Hashimura et al^29^ found delayed PP or thrombosis in 8 of 32 consecutive cases of single-layer stent CAS (25%) using computed tomography angiography or US within 1 week of CAS, with a minor stroke occurring in 1 of these 8 patients (12.5%). Moulakakis et al^30^ described a series of 674 CAS treated using conventional stents, with 4 cases (0.6%) showing acute thrombosis within 4 days of stenting. Those in-stent thromboses may have been associated with delayed PP.

Regarding the CRS, Imamura et al^31^ documented that among 140 CAS cases, 1 of the 2 strokes occurring within 30 days after CAS was associated with delayed PP on the cervical US. Okumura et al^32^ also reported a case of symptomatic in-stent occlusion 2 weeks after stenting with a CRS.

On the basis of those studies, delayed PP with CRS appears associated with an increased ischemic stroke risk within 30 days. In the present study, although delayed PP was observed in 3.4% of cases, stroke did not occur in any of the patients. We hypothesize that additional stent placement was effective in preventing acute stent occlusion due to delayed PP. Early detection of PP thus seems crucial in preventing ischemic stroke.

In the present study, all patients with delayed PP had unstable plaque, which is considered a risk factor for intraprocedural PP. Unstable plaque might also represent a risk factor for delayed PP. As a result, cases with unstable plaque stenosis should be carefully followed up using US even if intraprocedural PP is not evident. Our findings seem to indicate that physicians should pay attention not only to intraprocedural PP but also to delayed PP.

### Limitations

First, this study included only a moderate number of patients with a relatively short duration of follow-up. Larger series with a longer-term follow-up are necessary. Second, we did not perform quantitative evaluation of PP. Third, PP was evaluated by IVUS alone in the present study whereas OCT would provide greater sensitivity. However, OCT might be too sensitive for clinically relevant PP. Finally, although we used 1 physical modality to detect PP intraprocedurally and at 7 days (US), the resolution of Duplex US is smaller than that of IVUS. Further investigations with a larger number of cases and using higher-resolution imaging modalities to evaluate PP may be valuable.

## Conclusion

The US-determined incidence of intraprocedural PP with CRS CAS was 2.2%. Delayed PP developed in an additional 3.4% of cases by 1 week after CAS. Although CRS might reduce ischemic complications by preventing PP during CAS, careful follow-up is warranted to minimize the clinical risks associated with delayed PP.
